# Effects of Surface Inclination on the Vertical Loading Rates and Landing Pattern during the First Attempt of Barefoot Running in Habitual Shod Runners

**DOI:** 10.1155/2015/240153

**Published:** 2015-07-15

**Authors:** W. An, M. J. Rainbow, R. T. H. Cheung

**Affiliations:** ^1^Department of Rehabilitation Sciences, The Hong Kong Polytechnic University, Kowloon, Hong Kong; ^2^Department of Mechanical and Materials Engineering, Queen's University, Kingston, ON, Canada K7L 3N6

## Abstract

Barefoot running has been proposed to reduce vertical loading rates, which is a risk factor of running injuries. Most of the previous studies evaluated runners on level surfaces. This study examined the effect of surface inclination on vertical loading rates and landing pattern during the first attempt of barefoot running among habitual shod runners. Twenty habitual shod runners were asked to run on treadmill at 8.0 km/h at three inclination angles (0°; +10°; −10°) with and without their usual running shoes. Vertical average rate (VALR) and instantaneous loading rate (VILR) were obtained by established methods. Landing pattern was decided using high-speed camera. VALR and VILR in shod condition were significantly higher (*p* < 0.001) in declined than in level or inclined treadmill running, but not in barefoot condition (*p* > 0.382). There was no difference (*p* > 0.413) in the landing pattern among all surface inclinations. Only one runner demonstrated complete transition to non-heel strike landing in all slope conditions. Reducing heel strike ratio in barefoot running did not ensure a decrease in loading rates (*p* > 0.15). Conversely, non-heel strike landing, regardless of footwear condition, would result in a softer landing (*p* < 0.011).

## 1. Introduction

Running has become one of the most popular sport activities in the world [1], while running-related injuries affect many runners. A recent study revealed that 73.9% of marathon runners reported pain-related injuries during a running event [2]. Another review showed a high prevalence of injury incidence per 1000 hours of running in both novice and recreational runners [3]. It is accepted that repetitive loading with insufficient remodeling time causes overuse injuries [4].

**Figure 1:**
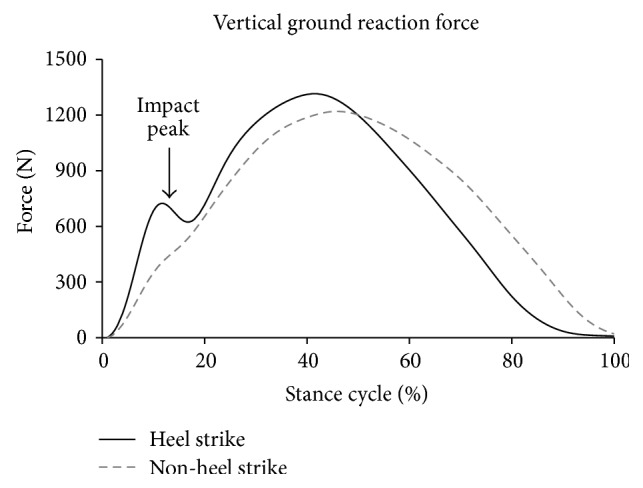
Vertical GRF of two steps extracted from one of the participants. In a heel strike landing, an impact peak existed, while, in a non-heel strike landing, the impact peak was diminished. When impact peak existed, it happened at around 13% of the total stance phase.

Vertical ground reaction force (VGRF) (Figure 1), defined as the vertical component of the force exerted by the ground onto the body, has been regarded as an important kinetic feature, where several injury-relevant parameters could be extracted [5, 6]. The average and peak rate at which the VGRF raises to its vertical impact peak (VIP), respectively, referred to as the vertical average loading rate (VALR) and vertical instantaneous loading rate (VILR), have been retrospectively associated with various running-related overuse injuries, such as tibial stress fractures [7–9] and plantar fasciitis [8]. As a result of these findings, several methods, such as gait pattern relearning strategies [10], landing pattern modification [11], and barefoot running [12], have been purported to lower injury risk by altering the kinetics during impact.

Although controversial, barefoot running has been proposed to be effective in lowering loading rates of VGRF [13]. A study comparing biomechanical differences between habitual shod and habitual barefoot runners suggested that barefoot running, through modulating landing pattern, decreases both VALR and VILR, compared with shod running [14]. In that particular study, habitual barefoot runners adopt a non-heel strike (NHS) landing pattern and are observed to sustain lower loading rates than shod runners who typically run with a heel strike (HS) landing pattern [14]. This could partially explain the growing prevalence of barefoot running amongst running communities [15]. The theory that barefoot running will naturally convert habitual shod, heel strike runners to a NHS pattern was partially supported by a recent study, in which novice barefoot runners who habitually landed with a HS pattern while shod converted to a mixed foot strike types (NHS and HS) within the first four minutes of barefoot running [5]. It is important to note that some runners persisted with a HS pattern while running barefoot and sustained high vertical loading rates.

Besides footwear, surface inclination is another factor to be cautiously inspected in running, which has been shown to alter running kinetics. Previous studies explored how the magnitudes of VIP and vertical active peak (VAP) differ during downhill and uphill running in a cohort of habitual shod runners [6, 16–18]. Their findings revealed that running downhill induced a significantly higher VIP and VAP than running uphill or on level ground. It has been shown that shod runners tend to have HS landing and NHS landing during downhill and uphill running, respectively. However, mechanical behavior during the first attempt of barefoot running on slope in habitual shod runners remains unknown.

Hence, the purpose of this study was to compare the landing pattern, VIP, VALR, and VILR from shod and barefoot running trials in three inclination conditions (inclined, declined, and level surface) amongst habitual shod runners. We hypothesized that running on declined treadmill would induce higher vertical loading rates than running on an inclined or level treadmill, regardless of footwear condition. We also hypothesized that early attempts of barefoot running would lead to a mixed landing pattern in habitual shod runners. However, running on declined treadmill may facilitate a HS landing while inclined treadmill running may lead to a NHS landing pattern.

## 2. Materials and Methods

Twenty runners (17 males; age = 28.5 ± 4.4 years; body mass = 69.2 ± 11.2 kg; body height = 1.74 ± 0.6 m) were recruited. All participants did not experience barefoot running or running with barefoot-simulating footwear prior to the experiment. They were free of symptoms for at least 6 months prior to recruitment. The institutional review board of the corresponding university had reviewed and approved the present study protocol. Written informed consent was obtained prior to participation in the study.

Before the experiment, all participants warmed up with low resistance cycling exercise for 10 minutes. During the running trials, participants ran with their usual running shoes and barefoot at 8.0 km/h on an instrumented treadmill (Advanced Mechanical Technology Inc., Watertown, MA, USA) at three different surface inclination angles (0°; +10°; −10°). All participants' running shoes were designed with a heel rise and medial arch support. Running trials were separated by a 15-minute rest period. The testing sequence of the footwear and slope conditions was randomized using an online program (https://www.random.org/). GRF was sampled at 1,000 Hz for 15 seconds after a 4-minute adaptation period. Participants' landing patterns were simultaneously examined by a high-speed camera (EX-F1, Casio, Tokyo, Japan) positioned near the ground level using a tripod and it was oriented perpendicular to the right side of the treadmill. The filming rate of the camera was set at 300 Hz. A HS pattern was identified when the right foot contacted the ground at any point within the rear one-third of the sole [19]. Otherwise, the footfall was regarded as a non-heel strike (NHS). The GRF data and landing images were synchronized using a customized LabVIEW program (National Instruments, TX, USA).

GRF data was filtered using a fourth order 50 Hz low-pass Butterworth filter and normalized by body mass [20]. VIP was identified as the impact transient that was generated when foot or shoe first contacted the ground [14]. In cases of absent impact transient, a set value of 13% stance was used as a surrogate for time to vertical impact peak [21]. VALR and VILR were obtained by the method described in Crowell and Davis [10] and Willy et al. [21]. In brief, VALR is the slope of the line through the 20% point and the 80% point of the 13% of the stance phase and VILR is the maximum slope of the VGRF in the same region. VALR and VILR were then normalized with stride length and were averaged across all footfalls in the observation period [22]. The landing pattern during shod and barefoot running was presented as a heel strike ratio (HS ratio), which was a ratio between the number of footfalls with a heel strike and the total number of contacts in the observation period. Therefore, a HS ratio of 100% indicates that all footfalls were heel strikes.

A 2 × 3 repeated measures ANOVA was used to compare HS ratio, VIP, VALR, and VILR in barefoot running and shod running against surface inclination angle (0°; +10°; −10°). In order to compare VALR and VILR in runners who did and did not reduce their HS ratio during barefoot running, a paired-sample *t*-test was conducted. A subgroup analysis was also performed using an independent-samples *t*-test in order to compare the loading variables between participants who adopt NHS in barefoot running (HS ratio = 0%), and those who totally or partially run with HS (HS ratio > 0%). Tukey's HSD was used for pairwise comparisons if applicable. Global alpha level was set at 0.05. All statistical analyses were conducted using PASW for Windows, version 18 (SPSS software, Chicago, IL, USA).

## 3. Results

We observed a significant difference in VIP, VALR, and VILR among surface inclination angles in shod running (*p* < 0.03) (Table 1). Pairwise comparisons indicated that declined treadmill running exhibited significantly higher VIP (Cohen's *d* = 0.69; *p* < 0.001; 95% CI: 0.20–0.72), VALR (Cohen's *d* = 0.49; *p* < 0.001; 95% CI: 27.07–92.19), and VILR (Cohen's *d* = 0.56; *p* < 0.001; 95% CI: 33.30–98.92), compared to level or inclined treadmill running. However, only VIP during barefoot running was influenced by surface inclination (*p* = 0.002). Similar to shod running, VIP during barefoot running was substantially greater in declined than in level or inclined condition (Cohen's *d* = 0.66; *p* = 0.002; 95% CI: 0.14–0.54). VALR and VILR during barefoot running did not differ across running slopes (*p* > 0.382).

A significantly lower HS ratio was found during barefoot running than shod running on level (Cohen's *d* = 0.94; *p* = 0.001), inclined (Cohen's *d* = 0.86; *p* = 0.002), and declined surfaces (Cohen's *d* = 0.97; *p* = 0.001) (Table 2) [23]. Conversely, we found that surface inclination was not a determinant for landing pattern, as the HS ratios at across different surface inclinations were similar (*p* > 0.413) (Table 2).

**Figure 2:**
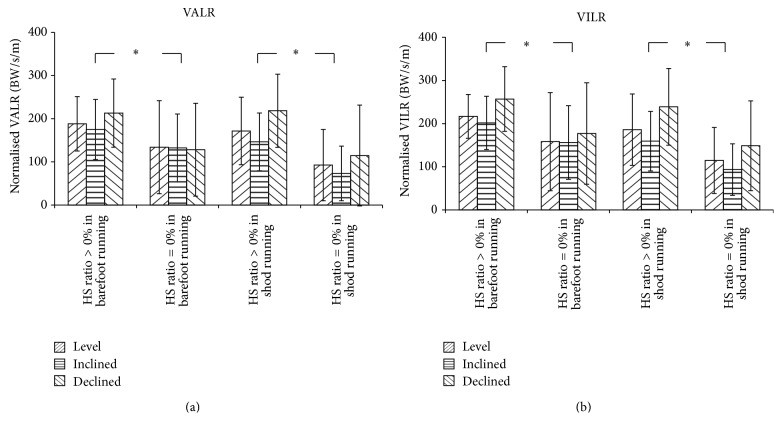
Comparison of VALR (a) and VILR (b) in barefoot and shod running between runners remained total or partial heels trike landing (HS ratio > 0%) and total non-heel strike landing (HS ratio = 0%), for three surface inclination angles. “*∗*” indicates that significantly higher (*p* < 0.05) loading rate was observed in HS ratio > 0% subgroup than in HS ratio = 0% under each footwear condition.

During the first attempt of barefoot running, most participants did not induce a complete transition from HS to NHS. Three participants, identified as forefoot or midfoot runners, adopted NHS throughout the experiment. Only one out of the remaining 17 subjects (5.88%) was observed to transition automatically from HS to NHS during the first attempt of barefoot running in all three surface inclinations. Runners who reduced their HS ratio during barefoot running compared to shod running under the same inclination condition (*n* = 14; trial = 39) did not show distinguished VALR (*p* = 0.60) or VILR (*p* = 0.15) between the two footwear conditions, neither did the runners who maintained or even increased the HS ratio (*n* = 7; trial = 11) (*p* = 0.95 for VALR; *p* = 0.39 for VILR) (Table 3). Runners who completely transitioned to NHS (*n* = 8, 9, and 7 for inclined, declined, and level treadmill running, resp.) in the barefoot condition had 31.41% lower VALR (Cohen's *d* = 0.72; *p* = 0.011; 95% CI: 14.53–105.66) and 26.47% lower VILR (Cohen's *d* = 0.69; *p* = 0.008; 95% CI: 16.07–102.45) than those who maintained complete or partial HS landing (Figure 2). The same phenomenon was also observed in shod condition (Cohen's *d* = 1.10, *p* = 0.007, 95% CI: 28.80–147.21 for VALR; Cohen's *d* = 0.98, *p* = 0.010, 95% CI: 22.44–135.24 for VILR).

## 4. Discussion

This study sought to examine the effects of surface inclination on the loading variables and landing pattern during the first attempt of barefoot running in habitual shod runners. We found that declined treadmill running would result in greater VALR and VILR than level and inclined treadmill running in shod, but not in barefoot, condition. However, we did not observe a significant difference of landing pattern at different slopes. We also noticed that nearly 95% of habitual shod runners did not completely transition to a NHS landing pattern during the first attempt of barefoot running on slopes. For runners who exhibited lower HS ratio during barefoot running than shod running under the same surface condition, their loading kinetics during barefoot running did not substantially differ from the ones during shod running. The same applied to those who ran with equal or higher HS ratio. However, VALR and VILR of runners who NHS were significantly lower than those who ran with HS or adopted a mixed landing pattern, regardless of their footwear condition.

High levels of VALR and VILR have been associated with running injuries. Previous biomechanical investigations on running kinetics mainly focused on level ground running. The insights provided by these studies may not apply to inclined or declined running. Nevertheless, most distance runners inevitably run on sloped terrain. Our findings were partially in accord with an antecedent study that examined running kinetics during downhill and uphill running [6]. Gottschall and Kram reported greater VIP during downhill running when comparing with level or uphill running. Their findings were in concordance with ours kinetically (Table 1). However, a transition from a pure heel strike landing (HS ratio = 100%) during downhill and level running to a mixed landing pattern (HS ratio < 100%) during uphill running was observed in Gottschall's study, whereas nearly half of our subjects (9 out of 20) exhibited a mixed landing pattern when running on declined or level treadmill in shod condition. Such discrepancy in observation should be due to the different runner groups that the two studies sampled. As previous studies indicated, both experienced barefoot and shod runners could adopt RFS, MFS, or FFS landing during running at 8 km/h [24, 25]. The participants we recruited consisted of RFS and non-RFS runners, which was different from the homogeneous RFS runner group in Gottschall's study. The difference in the exhibited landing pattern could therefore be appreciated.

Since the landing pattern was not strongly modulated by surface inclination, we speculated that the increased vertical loading rates during declined treadmill shod running could be a result of greater vertical displacement [26, 27] or increased joint stiffness [28, 29] or a combination of both factors. As opposed to our hypothesis, however, VALR and VILR did not show significant contrast across inclination conditions in novice barefoot runners. Previous studies demonstrated that barefoot running could either reduce leg stiffness from an instantaneous perspective [30] or increase it from an overall perspective [31]. A recent investigation into habitual shod runners during their first attempt of minimal shoes revealed that their vertical stiffness increased as inclination angle increased [32]. Moreover, runners exhibited higher leg stiffness when running with minimal shoes than regular shoes, regardless of surface inclination [32]. Considering that barefoot and minimalist running share similar lower limb kinematics [33, 34], such discrepancies could also be expected between barefoot and shod running. Therefore, the lower extremity mechanics embedded with shod and novice barefoot running, such as vertical and leg stiffness, could be intrinsically different, which might lead to different sensitivity of loading rates towards slope changes or nonuniformity of such sensitivity across subjects. The difference in pattern of loading rate change across inclination conditions between shod and first-time barefoot running could then be appreciated. However, the intrinsic reason for such difference still needs further exploration.

Most of the habitual shod runners, upon their first attempt of barefoot running, did not automatically and completely transition from HS to NHS landing on different surface inclination angles. No significant change in loading rate was observed neither in runners who reduced their HS ratio during barefoot running nor in those who did not. One possible explanation is that HS landing induces higher loading rates during barefoot than shod running, while NHS leads to lower loading rates [14]. Since some runners adopted mixed landing pattern throughout the barefoot running trials, the combination of such plus and minus effect could therefore generate results comparable to shod running. However, when complete NHS landing was isolated from HS and mixed landing patterns, the impact of NHS emerged. Running with NHS landing sustained lower VALR and VILR than with complete or partial HS landing, regardless of the footwear condition. Novice barefoot runners were therefore prone to experience high VALR and VILR before they managed to completely modify their landing pattern [14]. Besides, NHS landing did not further reduce the loading rates during barefoot than shod running, as observed in Lieberman et al. [14]. Difference in runners' experience in barefoot running could be the major reason behind this. Novice barefoot runner may sustain high leg stiffness, which leads to high loading rates [32, 35]. A recent study introducing a training method to reduce leg stiffness and loading rate in novice barefoot runners partially justified this point [36]. The high loading rate in novice barefoot runners with mixed landing pattern may be related to some of the injuries, such as metatarsal stress fractures and calcaneal stress fractures reported in novice barefoot runners [37–39].

In light of these findings, shod runners are encouraged to consult relevant professionals to ensure a safe and effective transition before they start barefoot running. Furthermore, transitioning to a complete NHS landing is more preferred than simply reducing HS ratio. An evaluation of landing pattern throughout a structured transition program is therefore suggested in order to reduce the risk of injury. In addition, shod runners who resume training from injury should avoid running downslope.

When interpreting our results it is important to consider several limitations in our study. First, we only tested the subjects at a constant and relatively slow speed. Therefore, findings of this study primarily concerned distance runners. Whether they are also applicable to sprinters needs further exploration. In addition, the inclination degree was relatively small. Future studies to investigate the biomechanical responses in runners running on a greater slope and different speeds are therefore warranted. Second, only 3 out of the 20 subjects recruited in this study were female. Previous studies reported a higher vertical loading rate in female runners compared to males [40], indicating a possible distinction between genders in kinematic or kinetic change when footwear or inclination condition varies. A participant group with more even sex ratio could enable a subgroup analysis on the aforementioned gender difference. Third, the duration of the running bout was short (4 minutes). It has been reported that muscle fatigue may affect running kinetics [41, 42]. The relationships between fatigue and landing pattern or vertical loading rates cannot be inferred from our results. Besides, the current HS identification method involved visual inspection, which was based on subjective judgment, and inevitably introduced extra error into the measurement. In future studies, advanced motion capture systems are recommended for more accurate landing pattern identification.

## 5. Conclusions

We found that declined treadmill running resulted in greater VALR and VILR than level and inclined treadmill running in shod condition, while such phenomenon was not observed during the first attempt of barefoot running. However, we did not observe a significant difference in the landing pattern between surface inclination angles. Simply reducing HS ratio during barefoot running did not guarantee a reduction in the loading rates. Completely adopting NHS landing should be the target of novice barefoot runners in order to ensure a safe transition. Most of the habitual shod runners did not completely transition to a non-heel strike landing upon their first attempt of barefoot running.

## Practical Implications

We have the following implications:

- Around 95% of habitual shod runners did not automatically alter their landing patterns to complete NHS during early exposure to barefoot running.
- Downslope running would result in greater VALR and VILR than level and upslope running in shod condition.
- Landing pattern may not differ among level, inclined, and declined treadmill running.
- Reducing HS ratio may not lead to reduction in the loading rates, while transition to complete NHS could likely induce it.

## Figures and Tables

**Table 1:** Comparison of vertical impact peak and vertical loading rates during inclined, level, and declined treadmill running.

	Inclined	Level	Declined	*p*
Shod running				
VIP (body mass)	1.21 ± 0.33^a,b^	1.51 ± 0.36^b,c^	1.78 ± 0.43^a,c^	<0.001^*∗∗∗*^
VALR (body mass/s/m)	131.73 ± 71.09^a,b^	159.72 ± 81.82^b,c^	202.95 ± 94.49^a,c^	0.030^*∗*^
VILR (body mass/s/m)	146.19 ± 70.99^a,b^	175.28 ± 83.88^b,c^	225.20 ± 94.17^a,c^	0.014^*∗*^
Barefoot running				
VIP (body mass)	1.24 ± 0.30^a,b^	1.39 ± 0.31^b,c^	1.65 ± 0.46^a,c^	0.002^*∗∗*^
VALR (body mass/s/m)	157.90 ± 74.27	169.16 ± 82.92	174.83 ± 100.08	0.820
VILR (body mass/s/m)	183.43 ± 73.56	196.17 ± 80.77	220.99 ± 102.09	0.382

Data are presented as mean ± standard deviation (SD).

^*∗*^ *p* < 0.05, ^*∗∗*^ *p* < 0.01, and ^*∗∗∗*^ *p* < 0.001.

a, b, and c indicate that the corresponding parameter is significantly different (*p* < 0.001) from the one in level, declined, and inclined condition, respectively.

**Table 2:** Comparison of landing pattern during barefoot and shod running under three surface conditions.

HS ratio (%)	Barefoot	Shod	*p*
Level	46.70 ± 43.61	84.27 ± 36.39	0.001^*∗∗*^
Inclined	32.28 ± 40.07	67.77 ± 42.80	0.002^*∗∗*^
Declined	37.92 ± 42.03	76.74 ± 37.51	0.001^*∗∗*^

*p*	0.558	0.413	

Data are presented as mean ± standard deviation (SD).

^*∗∗*^ *p* < 0.01.

**Table 3:** VALR and VILR for participants who exhibited a lower/higher HS ratio in barefoot running than in shod running under each inclination condition.

	Lower HS ratio in BF than in shod running				Higher HS ratio in BF than in shod running			
	Level	Inclined	Declined	Mean	Level	Inclined	Declined	Mean
*N*	11	14	14		6	2	3	

VALR (BW/s/m)								
Barefoot	206.78 (70.21)	184.43 (63.99)	196.98 (101.48)	195.24 (79.40)	157.50 (70.10)	106.59 (50.98)	186.89 (66.81)	156.26 (66.22)
Shod	178.51 (85.78)	154.66 (65.71)	221.90 (88.59)	185.53 (83.38)	158.84 (67.17)	88.24 (55.48)	202.83 (77.22)	158.00 (73.03)

VILR (BW/s/m)								
Barefoot	231.55 (68.36)	211.35 (60.49)	244.30 (101.31)	228.88 (78.71)	194.75 (52.53)	145.55 (17.37)	240.36 (52.28)	198.24 (55.24)
Shod	193.49 (91.09)	167.41 (68.80)	242.07 (93.66)	201.57 (88.64)	172.13 (69.72)	102.84 (48.96)	222.90 (75.30)	173.38 (74.40)

Data are presented as mean (standard deviation).
